# Biomarker Development Trial of Satraplatin in Patients with Metastatic Castration-Resistant Prostate Cancer

**DOI:** 10.1093/oncolo/oyac224

**Published:** 2022-12-15

**Authors:** Bobby C Liaw, Che-Kai Tsao, Sonia Seng, Tomi Jun, Yixuan Gong, Matthew D Galsky, William K Oh

**Affiliations:** Division of Hematology and Medical Oncology, The Tisch Cancer Institute, Icahn School of Medicine at Mount Sinai, New York, NY, USA; Division of Hematology and Medical Oncology, The Tisch Cancer Institute, Icahn School of Medicine at Mount Sinai, New York, NY, USA; Division of Hematology and Medical Oncology, The Tisch Cancer Institute, Icahn School of Medicine at Mount Sinai, New York, NY, USA; Sema4, Stamford, CT, USA; Division of Hematology and Medical Oncology, The Tisch Cancer Institute, Icahn School of Medicine at Mount Sinai, New York, NY, USA; Division of Hematology and Medical Oncology, The Tisch Cancer Institute, Icahn School of Medicine at Mount Sinai, New York, NY, USA; Division of Hematology and Medical Oncology, The Tisch Cancer Institute, Icahn School of Medicine at Mount Sinai, New York, NY, USA

**Keywords:** prostate cancer, satraplatin, biomarker, DNA damage, chemotherapy

## Abstract

**Background:**

In the phase III SPARC trial, satraplatin, an oral platinum analogue, demonstrated anticancer activity in men with metastatic castration-resistant prostate cancer (mCRPC). Repeat biopsies are uncommon in mCRPC, limiting the feasibility of tissue–based biomarkers. This phase II study sought to evaluate the feasibility and utility of blood–based biomarkers to identify platinum–sensitive mCRPC.

**Methods:**

Patients with mCRPC who had progressed on docetaxel were enrolled at a single center from 2011 to 2013. Subjects received satraplatin 80 mg/m^2^ by mouth daily on days 1-5 and prednisone 5 mg PO twice daily, on a 35-day cycle. Serial peripheral blood samples were collected for biomarker assessment.

**Results:**

Thirteen docetaxel-refractory mCRPC patients were enrolled, with a median age of 69 years (range 54-77 years) and median PSA of 71.7 ng/mL (range 0.04-3057). Four of 13 patients (31%) responded to satraplatin (defined as a PSA decline of ≥30%). Responders demonstrated improved time to disease progression (206 vs. 35 days, HR 0.26, 95% CI, 0.02-0.24, *P* = .003). A 6-gene peripheral blood RNA signature and serum tissue inhibitor of metalloproteinase-1 (TIMP-1) levels were assessed as biomarkers, but neither was significantly associated with response to satraplatin.

**Conclusion:**

In this small series, one-third of mCRPC patients responded to platinum–based chemotherapy. Peripheral blood biomarker measurement is feasible in mCRPC, though the biomarkers we investigated were not associated with platinum response. Other biomarkers, such as DNA damage repair mutations, should be evaluated.

## Teaser

Repeat biopsies are uncommon in metastatic castration–resistant prostate cancer (CRPC), limiting the feasibility of tissue–based biomarkers. This article evaluates the feasibility and utility of blood–based biomarkers to identify platinum–sensitive mCRPC.

**Lessons Learned**

- Peripheral blood biomarkers, including RNA expression and protein levels, can be measured and monitored in castration–resistant prostate cancer (mCRPC).
- More work is needed to identify biomarkers for platinum–based chemotherapy agents which have activity in mCRPC.

## Discussion

The natural history of prostate cancer can be long, yet repeat biopsies are uncommon. Blood–based biomarkers create an opportunity for dynamic reassessment and therapy selection. This has become increasingly relevant as more treatments and combinations have become available for castration–resistant prostate cancer (mCRPC). This study evaluated a whole blood 6–gene RNA transcript–based signature and serum tissue inhibitor of metalloproteinase-1 (TIMP-1) levels as biomarkers for response to the oral platinum analog satraplatin (Table 1).

**Table 1.** Demographics.

Number of patients	13
Age, years, median (range)	69 (54-77)
Baseline labs, median (range)	
PSA (ng/mL)	71.67 (0.04-3056.97)
Alkaline phosphatase (U/L)	93 (65-666)
Hemoglobin (g/dL)	11.9 (9.6-13.8)
White blood cells (×10^3^/µL)	6.7 (4.7-13.6)
Creatinine (mg/dL)	0.9 (0.68-1.83)
Gleason score, *n* (%)	
7	4 (31%)
8	5 (38%)
9	4 (31%)
Disease involvement, *n* (%)	
Bone metastases	12 (92%)
Lymph nodes	4 (31%)
Viscera	6 (46%)
Symptomatic, *n* (%)	
Yes	8 (62%)
No	5 (38%)
Prior therapy, *n* (%)	
Androgen deprivation therapy	13 (100%)
Docetaxel	13 (100%)
Cabazitaxel	4 (31%)
Mitoxantrone	2 (15%)
Abiraterone	5 (38%)
Enzalutamide	1 (8%)
Sipuleucel-T	1 (8%)
Ketoconazole	4 (31%)
Other antiandrogens	12 (92%)
Number of prior therapies, *n* (%)	
2 Lines	5 (38%)
3 Lines	1 (8%)
4 Lines	6 (46%)
5 Lines	1 (8%)

The 6–gene RNA score was previously validated to have prognostic significance in mCRPC. However, no relationship was observed between changes to the score and response to satraplatin. The biological processes measured by the 6–gene score may be distinct from those that determine response to satraplatin. A prognostic score may be more suited to identify patients who need treatment intensification rather than predicting benefits from a specific agent.

Tissue inhibitor of metalloproteinase-1 (TIMP-1) plays contradictory roles in tumorigenesis, opposing tumor invasion yet promoting cell growth and angiogenesis. Our data suggest that serum TIMP-1 levels may have some association with response to platinum therapy, as responders experienced an overall decrease in TIMP-1 levels, whereas non-responders had an overall increase. This is in line with observations that TIMP-1 levels are associated with neuroendocrine prostate cancer (NEPC), which is in turn associated with platinum sensitivity. Further evaluation is needed to evaluate the utility of trending TIMP-1 as a marker in NEPC.

Due to the withdrawal of support, this study of satraplatin did not meet the originally planned enrollment goal of 30 patients, which limits the analysis. The primary endpoint of PSA response rate in the evaluable cohort of patients in this study is comparable to the experience with satraplatin in the phase III SPARC trial. As expected, responders to satraplatin had a clear advantage in time to disease progression (206 vs. 35 days, HR 0.26, 95% CI, 0.02-0.24, *P* = .003).

In the time since the inception of this study, many new treatment options have become available for patients with mCRPC, and platinum chemotherapy has largely been limited to the treatment of NEPC. This study highlights a few issues which remain relevant: (1) platinum chemotherapy has activity in mCRPC but a biomarker for platinum sensitivity is needed, (2) the need for a platinum sensitivity biomarker overlaps with the need for a consensus clinical or molecular definition of NEPC, and (3) liquid biopsies permit dynamic molecular reassessment and could enable optimal treatment sequencing in mCRPC.

**Table:** 

Trial Information	
Disease	Prostate cancer
Stage of disease/treatment	Metastatic, castration–resistant
Prior therapy	Docetaxel
Type of study	Open-label, single-arm, phase II study
Primary endpoint	PSA response (≥30% decline from baseline, confirmed with a second level at least 3 weeks after)
Secondary endpoints	Time to disease progression, overall survival (OS), objective disease response by RECIST criteria, and safety. Safety outcomes included frequency and severity of adverse events and serious adverse events.
Investigator’s analysis	Active but results overtaken by other developments; correlative endpoints not met but clinical activity observed

## Additional Details of Endpoints or Study Design

### Participants

This open-label, single-arm, phase II study was performed at Mount Sinai Medical Center (New York, USA). Patient, were aged 18 or older, had histologically confirmed metastatic prostatic adenocarcinoma; castrate levels of testosterone (<50 ng/dL) on androgen deprivation therapy (ADT) with GnRH agonist to be continued while on study unless patient has previously undergone bilateral orchiectomy; discontinued antiandrogens 30 days prior to baseline PSA; progressed on at least one line of a prior docetaxel–based chemotherapy; had adequate organ and bone marrow function (ANC >1500/μL, platelets >100 000/μL, GFR >30 mL/min, AST and ALT ≤2.5x ULN or ≤5x ULN for patients with liver metastasis); had an ability to swallow oral medications whole; and had an Eastern Cooperative Oncology Group performance status of 0-2. Patients could have received other approved prostate cancer therapies.

Key exclusion criteria included prior treatment with platinum chemotherapy, uncontrolled intercurrent illness, medical contraindication to image–guided biopsies, history of prior malignancy (with the exception of adequately treated basal cell or squamous cell skin cancer or other cancers for which the subject has been disease-free for at least 5 years), and radiation therapy to ≥25% of the bone marrow within 30 days prior to being registered for protocol therapy.

The research protocol was approved by the Mount Sinai Institutional Review Board (New York, USA). All patients provided written informed consent.

### Procedures

Patients received treatment with satraplatin 80 mg/m^2^ PO daily, rounded to the nearest 10 mg dose, on days 1-5 of a 35–day treatment cycle. In addition, patients received prednisone 5 mg PO twice daily which was continued for as long as the patient received satraplatin on the clinical trial. Patients were allowed to continue satraplatin at the discretion of the investigator until objective or clinical disease progression, or until the occurrence of an unacceptable safety or tolerability issue. Continuation of satraplatin was contingent upon demonstrating an ANC ≥1500/μL, platelets ≥100 000/μL, and nonhematologic toxicities attributed to the study drug resolved to baseline or grade ≤1 (except alopecia) or grade ≤2 for pain. Up to 2 dose delays or reductions of satraplatin were allowed (80-60 mg/m^2^ daily, 60-40 mg/m^2^ daily).

Patients were discontinued from the study if they demonstrated PSA progression while on satraplatin, defined as a ≥25% PSA increase from either nadir or pretreatment value (whichever is lower) and a minimum absolute PSA increase by ≥2 ng/mL. Patients had to have been on treatment for at least 2 cycles in order to be evaluable for PSA progression, and PSA values had to be confirmed at least 3 weeks later. Other grounds for discontinuation of study were the development of significant adverse events or toxic effects, where continued administration of the study drug was deemed not to be in the patient’s best interest.

Follow-up visits for evaluation and safety checks were scheduled to coincide with the start of each 35–day cycle. Blood samples to establish serum PSA, blood count, renal and hepatic function were collected at screening, on day 1, and every 5 weeks at the start of each new treatment cycle. Radiologic studies (CT or MRI of chest, abdomen, and pelvis, plus bone scan) to evaluate for interval changes in disease were performed every 2 treatment cycles.

As part of scientific correlative endpoints, serum as well as whole blood in PAXgene Blood RNA tubes were collected at the time of enrollment (pre-treatment), prior to cycle 10 (on-treatment), and upon progression.

### Outcomes

The primary clinical endpoint of this study was PSA response rate as outlined by the Prostate Cancer Clinical Trials Working Group 2 (PCWG2).^1^ PSA response was defined in this trial as a ≥30% decline from baseline, confirmed with a second level at least 3 weeks after, calculated as the percentage change in PSA level from baseline, as well as the maximum decline in PSA that occurs at any point during treatment. Patients who did not complete at least 2 cycles of therapy were not evaluable for PSA or overall response.

Secondary endpoints included time to disease progression, overall survival (OS), objective disease response by RECIST criteria, and safety. Safety outcomes included frequency and severity of adverse events and serious adverse events.

Exploratory endpoints included a correlation of response to satraplatin with changes to a whole blood RNA 6–gene signature score^2^ and serum TIMP-1 levels.

### Procedures

Whole-blood samples were collected in PAXgene Blood RNA tubes and processed to total RNA with the Qiagen PAXgene Blood RNA Kit (Qiagen, Valencia, CA, USA). Target-gene amplification was done in a quantitative PCR reaction with TaqMan Universal PCR Master Mix (Applied Biosystem, Division of Life Technologies Corporation, Carlsbad, CA, USA) and Precision Profiles (Source MDx, Boulder, CO, USA). Individual target-gene amplification was multiplexed with the eukaryotic 18S rRNA endogenous control and run-in triplicate in a 384-well format on the 7900HT fast real–time PCR system. Normalized CT values (ΔCT) for each amplified target gene replicate were calculated. Resulting in triplicate ΔCT values for individual target genes were averaged yielding a final ΔCT value. The 6–gene score was calculated based on the model: 2×ABL2+SEMA4D+ITGAL–C1QA–TIMP1–CDKN1A. Maximum change to each patient’s 6–gene score while on treatment was compared to baseline values and recorded as a percentage change.

Peripheral blood samples were collected, and serum was separated out for testing using the TIMP-1 and TIMP-2 Duoset ELISA kit (R&D Systems) according to manufacturer instructions. Serum samples were diluted 400-fold (1 μL diluted in 399 μL of reagent diluent), and a standard curve was run along with the serum samples. Optical density is measured on a plate reader at 450 nm.

### Statistical Analysis

All patients evaluable for response to satraplatin were characterized as responders or non-responders. Kaplan–Meier curves were generated for each of the 2 groups and the differences between the 2 groups were assessed using the log–rank test.

**Table:** 

Drug Information	
Generic/working name	Satraplatin
Company name	GPC Biotech AG
Drug type	Chemotherapy
Drug class	Platinum-based
Dose	80
Unit	mg/m^2^
Route	Oral
Schedule of administration	Daily for days 1-5 of a 35–day cycle

**Table:** 

Patient Characteristics	
Number of patients, male	13
Number of patients, female	0
Stage	mCRPC
Age: median (range)	69 (54-77) years
Number of prior systemic therapies: median (range)	4 (2-5)
Performance status: ECOG	0:4 1:8 2:1 3:0 4:0
Cancer types or histologic subtypes	Prostate cancer, 13; Gleason 7, 4; Gleason 8, 5; Gleason 9, 4; Neuroendocrine features, 2

**Table:** 

Primary Assessment Method	
Number of patients enrolled	13
Number of patients evaluable for toxicity	13
Number of patients evaluated for efficacy	10
Evaluation method	Tumor marker

## Outcome Notes

Primary endpoint analysis of the PSA response rate demonstrated that 31% (4/13) were considered responders to satraplatin, with a ≥30% reduction in PSA as compared to baseline levels. A total of 46% (6/13) were considered non-responders.

There was a significantly improved time to disease progression, defined by PSA and/or radiographic progression, in responders as compared to non-responders (206 vs. 35 days, HR 0.26, 95% CI, 0.02-0.24, *P* = .003). A trend towards improvement in OS was suggested in those with a response to satraplatin therapy but was not found to be statistically significant (570 vs. 486 days, HR 0.50, 95% CI, 0.097-1.997, *P* = .322).

**Table:** 

Secondary Assessment Method	
Number of patients enrolled	13
Number of patients evaluated for efficacy	10
Evaluation method	RECIST 1.1; time to disease progression and overall survival
Response assessment, PR	1 (10%)
Response assessment, SD	9 (90%)
Median duration assessments, TTP	36 days (CI, 34-194)
Median duration assessments, OS	513 days (CI, 459-644)

## Outcome Notes

The objective response rate was 10%, with 1 partial response and 9 patients with stable disease. There was a significantly improved time to disease progression, defined by PSA and/or radiographic progression, in responders as compared to non-responders (206 vs. 35 days, HR 0.26, 95% CI, 0.02-0.24, *P* = .003). A trend towards improvement in OS was suggested in those with a response to satraplatin therapy but was not found to be statistically significant (570 vs. 486 days, HR 0.50, 95% CI, 0.097-1.997, *P* = .322).

### Six-Gene Whole Blood RNA Score

**Figure 1.**
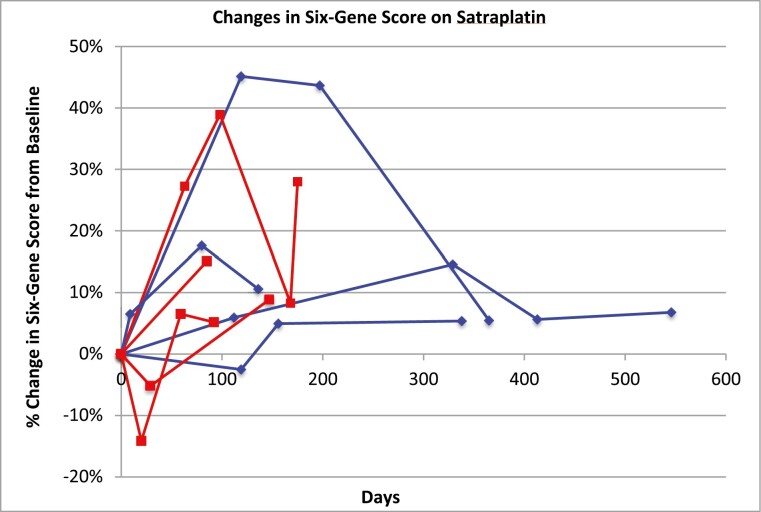
Changes in individual patients’ 6–gene signature scores over the course of satraplatin treatment. Responders (achieving ≥30% decline in serum PSA) are shown in blue, non-responders shown in red.

Four responders and 4 non-responders had paired pre- and post-treatment samples available for peripheral blood transcriptional profiling. No clear pattern of change was noted in the 6–gene signature scores in both the responder and non-responder groups (Fig. 1).

**Figure 2.**
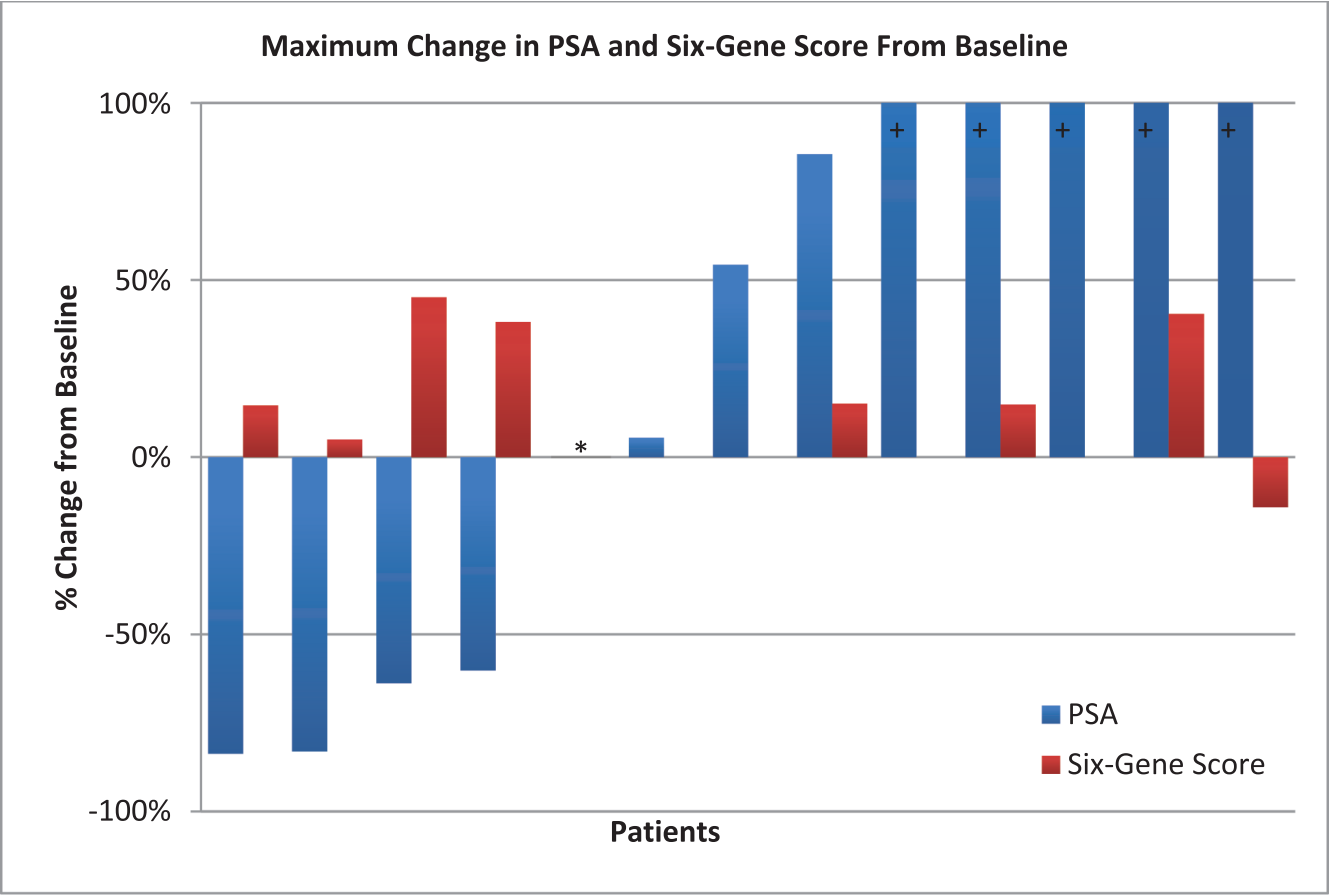
Maximum changes in PSA levels (blue) displayed as a waterfall plot, with associated 6–gene signature score changes (red) displayed adjacently. The 6–gene scores were calculated for patients that tolerated at least 2 cycles of satraplatin treatment. (+) denotes a PSA change >100%. (*) no follow up PSA labs available for comparison to baseline PSA.

Maximum percentage change to each patient’s 6–gene score was compared to the maximum percentage PSA change and visualized in a paired waterfall plot (Fig. 2). All 4 responders to satraplatin had increased to their 6–gene score despite decreases in their PSA levels, whereas the non-responders had variable changes.

### Serum TIMP-1 Levels

**Figure 3.**
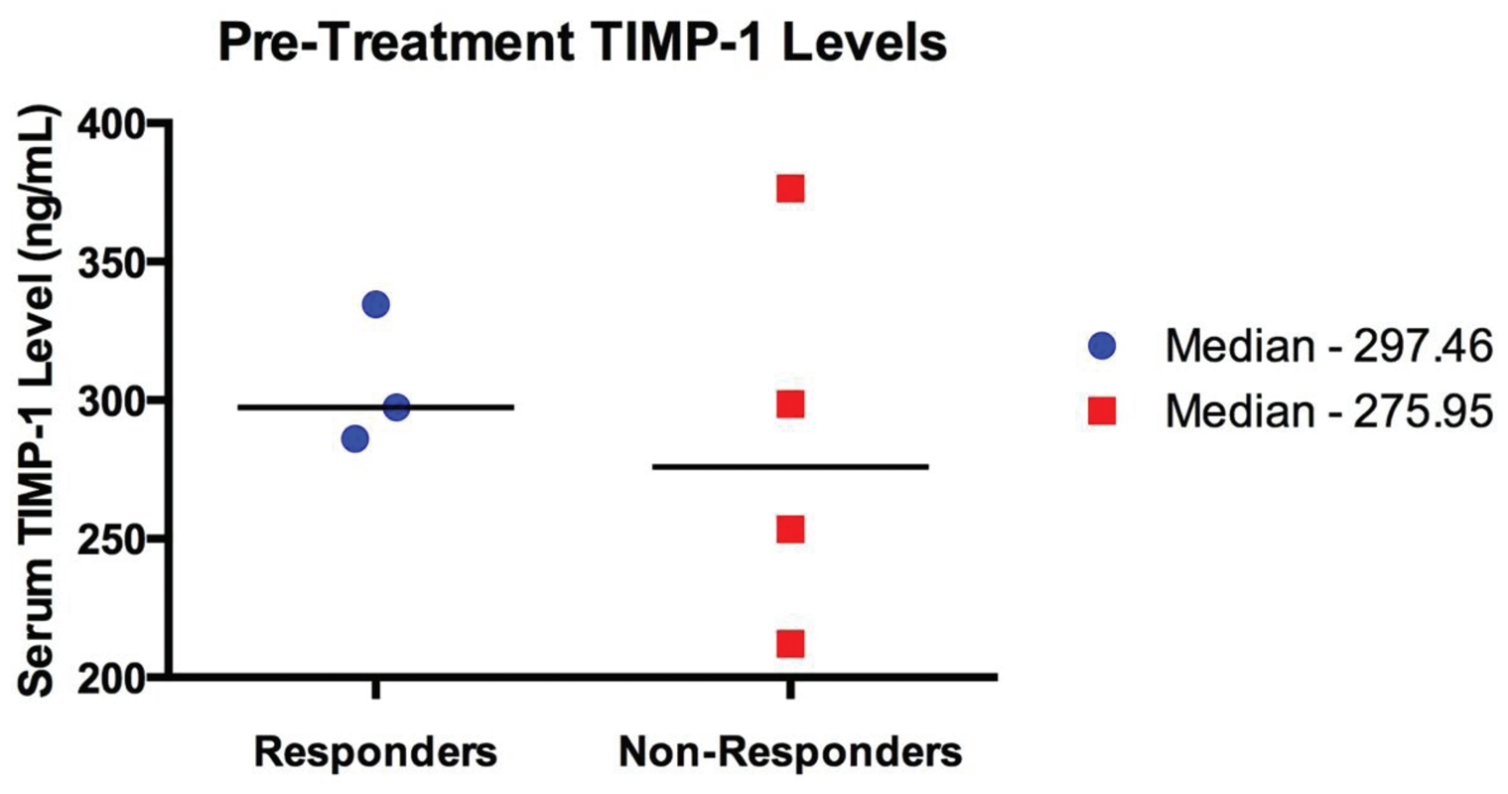
Baseline pre-treatment serum TIMP-1 protein levels in satraplatin responders and non-responders. There is no significant difference in the medians between the 2 groups, *P* = .86. Only patients with paired pre- and post-treatment blood samples had serum TIMP-1 levels tested by ELISA.

Three responders and 4 non-responders had sufficient paired pre- and post–treatment samples available for serum TIMP-1 level testing by ELISA. Median baseline serum TIMP-1 levels were 297.5 ng/mL in the responder group and 276.0 ng/mL in the non-responders (*P* = .86) (Fig. 3). There was more variation in the baseline serum TIMP-1 levels in the non-responders, ranging 212 to 376.44 ng/mL, whereas the responders were within a narrower range, 286.14 to 334.53 ng/mL.

All 3 responders demonstrated decreases in their TIMP-1 levels following the initiation of satraplatin, with a median decrease of 20.9%. One non-responder had a decrease in TIMP-1 levels after starting satraplatin, but as a group, the non-responders had a median increase of 16.1% in TIMP-1 levels (*P* = .40).

**Table:** 

Assessment, Analysis, and Discussion	
**Completion**	**Study completed**
Investigator’s assessment	Active but results overtaken by other developments; correlative endpoints not met but clinical activity observed

Platinum chemotherapy has definite antineoplastic activity in metastatic prostate cancer,^3^ but given modest rates of response, predictive biomarkers are needed to enrich for optimal patients to receive therapy. Historical trends suggest increasing rates of non-osseous metastatic disease,^4^ implicating neuroendocrine differentiation as a possible contributing factor, for which platinum agents remain a treatment option. Clinical activity for platinum compounds has also been strongly correlated to cancers with DNA repair abnormalities.^5^ Widespread adoption of next-generation genomic profiling has uncovered enrichment of mutations in homologous recombination DNA repair genes and DNA damage checkpoints in more advanced stages of the disease, including genes such as BRCA1, BRCA2, ATM, CHEK2, PALB2, RAD51D,^6,7^ suggesting that they are acquired during progression or in response to prior lines of therapy. This also lays a clear rationale for revisiting platinum chemotherapy in mCRPC.

Due to the withdrawal of support, this study of satraplatin did not meet the originally planned enrollment goal of 30 patients, which limits the analysis. The primary endpoint of PSA response rate in the evaluable cohort of patients in this study is comparable to the experience with satraplatin on the phase III SPARC trial: 31% of study patients (≥30% PSA decline) vs. 25.4% in SPARC (≥50% PSA decline) (Table 2).^8^ By implementing the ≥50% PSA decline definition for PSA response used by SPARC, this study would have an adjusted 23% PSA response rate comparable to SPARC.

**Table 2.** Response to satraplatin treatment.

Number of cycles received	
Median	3
Range	1-15
Number of dose reductions	
Median	0
Range	0-4
Response to treatment, *n* (%)	
Responders	4 (31%)
Non-responders	6 (46%)
Not evaluable	3 (23%)
Time to disease progression, days, median (range)	
Responders	194 (73-356)
Non-responders	34 (34-36)
Overall	36 (34-194)
Overall survival, days, median (range)	
Responders	570 (482-717)
Non-responders	459 (419-644)
Overall	513 (459 - 644)
Time to PSA nadir, days, median (range)	
Responders	165.5 (68.0%-236.0%)
PSA change at nadir, days, median (range)	
Responders	73.4% (60.2%-83.7%)

The median time to disease progression of 5.5 weeks on study is noticeably short, especially as compared to the 11.1 weeks observed in the satraplatin arm of SPARC,^6^ but our cohort of patients received more extensive pretreatment prior to enrollment on study. The majority of participants on study received at least one line of AR–targeted therapy (abiraterone, enzalutamide) and/or second–line chemotherapy (cabazitaxel). Median OS of 73.3 weeks in this overall study population is slightly higher than that reported by SPARC (61.3 weeks),^8^ our analysis excluded patients who received less than 2 cycles.

As expected, responders to satraplatin had a clear advantage in time to disease progression (206 vs. 35 days, HR 0.26, 95% CI, 0.02-0.24, *P* = .003). Radiographic evaluation found that all 4 responders to satraplatin therapy had stability of their disease by RECIST criteria following 2 cycles of treatment. One responder subsequently developed radiographic progression by CT on day 218 of follow-up, prior to any evidence of PSA progression. Another responder had stable disease on serial imaging studies for the first 15 cycles of treatment and subsequently was found to have a delayed partial response by CT. All non-responders demonstrated progression of PSA before any evidence of radiographic progression. The small sample size limits our analysis, but a numerical improvement in median OS was observed in satraplatin responders (570 vs. 486 days, HR 0.50, 95% CI, 0.097-1.997, *P* = .322), though not statistically significant. Adverse events are shown in Table 3.

**Table 3.** Incidence of most common adverse events.

Adverse event	Patients	Percentage(%)	Grade 1	Grade 2	Grade 3	Grade 4	Grade 5	Not graded
Alopecia	3	23	3					
Anemia	2	15		2				
Anorexia	5	38	3	2				
Anxiety	3	23	3					
Chronic back pain	1	8		1				
Confusion	1	8						1
Constipation	7	54	3	3	1			
Cough	2	15	2					
Cystitis	1	8		1				
Decreased appetite	1	8	1					
Dehydration	1	8						1
Depression	4	31	4					
Diarrhea	5	38	4		1			
Dysphagia	1	8						1
Dyspnea	4	31	2		2			
Dysuria	1	8						1
Edema	3	23	3					
Edema-lower extremity	2	15	1					1
Fatigue	10	77	6	3		1		
Fever	1	8	1					
Hematuria	2	15	2					
Hyperkalemia	1	8	1					
Hypocalcemia	2	15	2					
Hypoxia	1	8						1
Increased ALT	1	8	1					
Increased AST	1	8	1					
Increased nocturia	1	8	1					
Increased urinary frequency	1	8		1				
Influenza	1	8						1
Insomnia	5	38	5					
Left pelvic pain	2	15		1	1			
Leukopenia	6	46	3		3			
Nasal congestion	1	8	1					
Nausea	4	31	2	2				
Neuropathy	2	15	2					
Neuropathy-feet, bilateral	2	15	2					
Neuropathy-right hand	1	8	1					
Neutropenia	4	31	1	2	1			
Nocturia	2	15	1	1				
Pain-abdomen	1	8		1				
Pain-abdomen, bilateral lower quadrant	1	8	1					
Pain-abdomen, right upper quadrant	1	8	1					
Pain-back	1	8			1			
Pain-disease-site	8	6	5	2	1			
Pain-leg, right	1	8	1					
Pain-pelvic, bilateral	1	8		1				
Pain-rib, left	1	8			1			
Pain-shoulder, right	2	15		2				
Pain-tooth	1	8	1					
Rash	1	8	1					
Renal failure	1	8					1	
Slow urine flow at night	1	8						1
Strain-right shoulder	1	8	1					
Stroke	1	8		1				
Tachycardia	1	8						1
Tachypnea	1	8						1
Thrombocytopenia	6	46	4	1	1			
Tightness-back, upper	1	8	1					
Urinary frequency	3	23	2					1
Vomiting	1	8	1					
Weak voice	1	8	1					
Weakness	5	38	3	1	1			
Weight loss	3	23	1	2				

Additional predictive markers for platinum sensitivity outside of genomic profiling need to be developed. Peripheral blood cells are altered by their interactions with neoplastic and stromal tissue, lending clinical value to peripheral blood genomic signatures. A whole blood 6–gene RNA transcript–based signature developed by our group has been validated to have prognostic significance for overall survival in mCRPC.^9^ The predictive capabilities of this 6–gene signature and how it changes in response to therapy are the subject of further evaluation, although data from our group suggest that the disease state and choice of therapeutic agent may impact the 6–gene score.^10^

Differences in peripheral blood profiling among responders and non-responders can be identified and used to facilitate biomarker development. However, in this study, no clear correlation was observed between changes to the 6–gene score and response to satraplatin. This may possibly be due to the 2 tests evaluating 2 distinct biological processes. PSA change and expression are regulated by AR–dependent biochemical pathways, whereas the genes that comprise the 6–gene RNA signature (ABL2, SEMA4D, ITGAL, C1QA, TIMP-1, CDKN1A) serve immunogenic functions, playing roles in regulating T-cell motility, antigen surveillance, B-cell activation, and macrophage differentiation.^9^

Apart from the 6–gene score, increased TIMP-1 expression itself has been observed in multiple tumor types in both solid and hematologic malignancies and plays contradictory roles in tumorigenesis. It has been shown to the inhibit degradation of extracellular matrix by matrix metalloproteinases (MMPs), which modulates tumor invasion and progression of metastatic disease,^11-13^ but has also been found to inhibit apoptosis,^14,15^ as well as promote cell growth and angiogenesis.^16-18^

Data from this study suggest that changes in serum TIMP-1 levels (measured by ELISA) may have some association with response to platinum therapy, as responders experienced an overall decrease in TIMP-1 levels, whereas non-responders had an overall increase. This is in line with previously published findings that higher levels of TIMP-1 have a greater association with neuroendocrine disease in prostate cell lines,^19^ and that elevated TIMP-1 expression is a significant independent prognostic factor for decreased survival in mCRPC.^2^ Further evaluation is needed to evaluate the utility of trending TIMP-1 as a marker in NEPC, as well as to see if elevated TIMP-1 levels correlate with the presence of DNA repair gene mutations.

Since DNA repair mutations are not exclusive to early-onset and histologically aggressive disease^20^ and there is not clear consensus on how to define neuroendocrine prostate cancer,^21,22^ a wide net of biomarkers should be investigated. Studies to establish the activity of platinum therapy at multiple different stages of prostate cancer are ongoing. Currently, it remains unclear when platinum agents should be introduced and how they should be sequenced, even if there is confirmed neuroendocrine differentiation or the presence of DNA repair mutations. Recent trials of PARP inhibitors have confirmed that targeting DNA repair defects has therapeutic value as well,^23,24,25^ and so biomarkers developed for PARP inhibitors may overlap in predicting platinum sensitivity, given the similarities in their mechanisms of action.

## Data Availability

The data underlying this article will be shared on reasonable request to the corresponding author.
